# Antibiotic utilization trends in Veterans Affairs patients with *Stenotrophomonas maltophilia* bloodstream infections

**DOI:** 10.1017/ash.2024.364

**Published:** 2024-09-09

**Authors:** Clara H. Lee, Ursula C. Patel, Amanda Vivo, Lishan Cao, Charlesnika T. Evans

**Affiliations:** 1 Department of Pharmacy, San Francisco VA Health Care System, San Francisco, CA, USA; 2 Department of Pharmacy, Edward Hines, Jr. VA Hospital, Hines, IL, USA; 3 Center of Innovation for Complex Chronic Healthcare, Edward Hines, Jr. VA Hospital, Hines, IL, USA; 4 Department of Preventive Medicine, Center for Health Services and Outcomes Research, Northwestern University Feinberg School of Medicine, Chicago, IL, USA

## Abstract

**Objective:**

*Stenotrophomonas maltophilia* is a multidrug-resistant gram-negative bacillus that can cause serious infections but has limited treatment options. This study aims to establish trends in the treatment of *S. maltophilia* bloodstream infections (BSI) across the United States in Department of Veterans Affairs (VA) facilities.

**Methods:**

Data was evaluated over a 10-year timeframe (2012 to 2021) in this retrospective cohort study. Veterans with ≥ 1 blood culture with *S. maltophilia* within a VA medical encounter were included. Microbiology, pharmacy, and patient information were collected through national VA data sources and chart review. Descriptive statistics and Poisson regression were used to summarize patient demographics, facility characteristics, microbiologic data, and treatment trends.

**Results:**

A total of 374 blood cultures positive for *S. maltophilia* were identified across 75 VA facilities. Of 282 unique patients with BSI, the majority were male (93.6%), white (67.4%), with a mean age of 64 ± 13.1 years. Of those patients, 78% received treatment, 12.8% had a polymicrobial blood culture, and 5.3% had a documented sulfa allergy. Susceptibility results were most reported for trimethoprim-sulfamethoxazole (TMP-SMX), levofloxacin, and ceftazidime, with 4.5%, 4.3%, and 44.4% resistant isolates, respectively. Antibiotics most prescribed included TMP-SMX (41.5%) and levofloxacin (39.4%), followed by ciprofloxacin (13.8%) and ceftazidime (12.4%). Combination therapy was prescribed in 33% of patients. No significant trends were found with antibiotic utilization over time.

**Conclusions:**

TMP-SMX and levofloxacin were the most prescribed antibiotics for *S. maltophilia* BSI treatment. No significant changes were seen with antibiotic prescribing trends in Veterans from 2012 to 2021.

## Introduction

*Stenotrophomonas maltophilia* is now a prevalent carbapenem-resistant gram-negative bacteria causing bloodstream infections (BSI) in the United States.^1,2^ While *S. maltophilia* is generally considered a pathogen with low virulence, opportunistic infections in susceptible patient populations can be problematic due to its intrinsic resistance to a wide range of antibiotics.^3^ Moreover, the incidence of *S. maltophilia* is thought to be increasing especially in immunocompromised patients and those exposed to broad-spectrum antibiotics.^3,4^ *S. maltophilia*, along with other gram-negative organisms, pose a threat to the Veteran population who often have comorbidities predisposing them to resistant pathogens. Risk factors for mortality in hospitalized patients with *S. maltophilia* include common chronic conditions such as arterial hypertension, type 2 diabetes, acute myocardial infarction, presence of a urinary catheter, hemodialysis, or peritoneal dialysis.^2^

Despite the potential for the pathogenicity of *S. maltophilia*, a paucity of scientific evidence on antimicrobial treatment options and effectiveness exists. As with many multidrug-resistant organisms, the treatment for *S. maltophilia* is not clearly defined. Traditionally, trimethoprim-sulfamethoxazole (TMP-SMX) has been used most frequently to treat infections associated with *S. maltophilia.* ^3,5^ However, this practice is largely based on in vitro studies, retrospective non-randomized clinical trials, or expert opinion, and there is limited evidence to support TMP-SMX as the preferred therapy along with optimal dosing to maximize its benefits.^2,5,6^ There have also been increasing concerns for TMP-SMX resistance, leading to questions about whether TMP-SMX would continue to be as effective against *S. maltophilia* as it was once thought to be.^7–9^

The Infectious Diseases Society of America’s (IDSA) suggested approach for the management of *S. maltophilia* infections has evolved since its initial release in 2021.^10^ Recent guidance focuses on moderate to severe cases of *S. maltophilia* infections with two main suggested approaches to management: either the combination of two potentially active agents, with options including TMP-SMX, minocycline or tigecycline, levofloxacin, or cefiderocol; or the combination of ceftazidime-avibactam plus aztreonam in the setting of significant clinical instability and no other viable options. Transitioning to monotherapy can be considered with appropriate clinical response, except for levofloxacin which is advised to be only used as a component of combination therapy due to concerns for resistance. At this time, the authors of the guidance document acknowledge that there is insufficient data to recommend a “standard-of-care” therapy, and there is a need for more robust clinical trials that compare the effectiveness of different treatment strategies and their impact on patient outcomes.

Little data exists on treatment trends for *S. maltophilia* infections, especially in the Veteran patient population, where older age and existence of multiple comorbidities increase this population’s vulnerability for infection and poor outcomes. The goal of this study is to describe antibiotic treatment strategies utilized to treat *S. maltophilia* BSI in the Veteran patient population to gain a better understanding of treatment approaches.

## Methods

This retrospective cohort study was conducted across all Department of Veterans Affairs (VA) healthcare facilities during a 10-year timeframe from January 2012 to December 2021. Data were extracted from the VA Corporate Data Warehouse (CDW), a national repository that includes clinical and administrative data from the Veterans Health Administration. These data were used to obtain microbiology, pharmacy, and encounter data, including facility characteristics, culture data, and antimicrobial treatment information. Additionally, patient demographics, characteristics, and comorbidities were also collected. Comorbidities were identified by ICD-10 codes in the last 365 days prior to an *S. maltophilia* culture and used to calculate the Charlson comorbidity index.^11^ Chart reviews were conducted to verify antibiotic treatment and microbiologic results and obtain additional patient demographic and medical data, including past medical history and allergy information.

Veterans were included if they were aged ≥ 18 years with ≥ 1 positive blood culture for *S. maltophilia* in an inpatient setting. Index date was defined as the collection date of the blood culture positive for *S. maltophilia*. Antimicrobial treatment was defined as administration of at least one agent with possible activity against *S. maltophilia* between days –2 and +5 of the index date. Antibiotics with possible activity against *S. maltophilia* for which data was collected included cefiderocol, ceftazidime, ceftazidime-avibactam plus aztreonam, ciprofloxacin, eravacycline, levofloxacin, minocycline, moxifloxacin, polymyxins, tigecycline, and TMP-SMX. Given broad-spectrum antibiotic exposure is a potential risk factor for *S. maltophilia* infections, data for antibiotic history was obtained by collecting documented administration of common broad-spectrum antibiotics 90 days prior to the index date. Microbiologic data collected included susceptibility results, if reported, identification of *S. maltophilia* in specimens other than blood, and whether the positive blood culture was monomicrobial or polymicrobial. Susceptibility results for blood cultures were collected based on textual interpretations (ie, resistant, intermediate, or susceptible) of the reported site.

Descriptive statistics were used to summarize patient demographics, medical characteristics, previous healthcare exposure, facility characteristics, microbiologic data, and antibiotics received. Poisson regression was applied as a trend test to assess changes in antibiotic usage over years. Two-sided *p*-values <0.05 were considered significant. Statistical analyses were conducted using SAS, version 9.2 (SAS Institute).

## Results

A total of 374 blood cultures positive for *S. maltophilia* were identified across 75 of the 171 VA facilities. Of these, 282 unique BSI cases were identified (Table 1) with 14% being polymicrobial. Among the 282 unique patients, the majority were male (93.6%), white (67.4%), with a mean age of 64 ± 13.1 years. Severity of comorbidities was assessed using the Charlson comorbidity index. The mean and median score of the 282 patients was 4, indicating a moderate to severe burden of comorbidities and increased risk of short-term mortality. Apart from older age, end-stage renal disease was a common comorbidity identified with 25.5% patients diagnosed with this at the time of *S. maltophilia* BSI. The most commonly administered antibiotics within 90 days prior to culture date were beta-lactam/beta-lactamase inhibitors (40.8%), third- or fourth-generation cephalosporins (38.9%), and fluoroquinolones (34%). The majority of the cases received care in Southern locations (46.1%) and close to 20% of all case encounters occurred in rural areas. Most patients were managed at higher complexity VA facilities.

**Table 1.** Patient and facility characteristics associated with *S. maltophilia* bacteremia

Patient characteristics	N = 282
Age, y, mean ± SD	64, 13.1
Age ≥ 65 years, median (IQR)	72 (68, 80)
Male, n (%)	264 (93.6)
Race/ethnicity, n (%)	
White	190 (67.4)
Black	73 (25.9)
Other	4 (1.4)
Missing	15 (5.3)
Ethnicity, n (%)	
Hispanic/Latinx	28 (9.9)
Non-Hispanic/Latinx	254 (90.1)
Clinical severity	
Charlson comorbidity index, mean ± SD	4 ± 2.9
Charlson comorbidity index, median (min-max)	4 (0–12)
Documentation of sulfa allergy, n (%)	15 (5.3)
End-stage renal disease, n (%)	72 (25.5)
Polymicrobial blood culture, n (%)	36 (12.8)
Specimens other than blood positive on same culture date, n (%)	
Pulmonary	8 (2.8)
Urine	2 (0.7)
Other	3 (1.1)
Antibiotic use (90 days prior), n (%)	
β-lactam/β-lactamase inhibitors	115 (40.8)
Cephalosporins	107 (37.9)
Fluoroquinolones	96 (34.0)
Carbapenems	54 (19.2)
Tetracyclines	23 (8.2)
Trimethoprim-sulfamethoxazole	22 (7.8)
Facility characteristics, n (%)	
Rural	56 (19.9)
Region	
Northeast	40 (14.2)
Northwest	53 (18.8)
South	130 (46.1)
West	48 (17.0)
Other	11 (3.9)
Complexity, n (%)	
1a-1c	274 (97.2)
2	5 (1.8)
3	3 (1.0)

SD, Standard deviation; IQR, Interquartile range.

**Figure 1.**
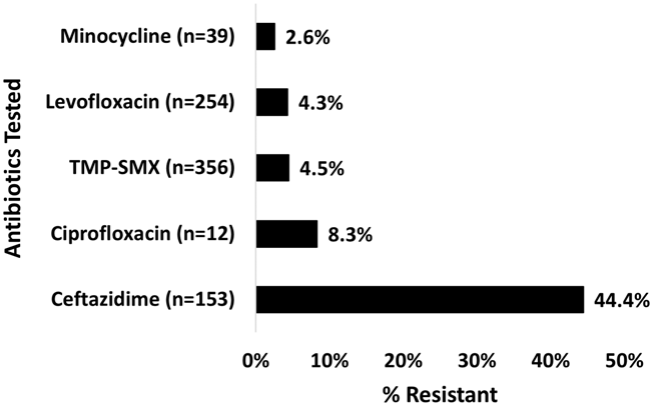
Frequency of resistant *S. maltophilia* isolates to commonly tested antibiotics.

Out of the 374 blood cultures positive for *S. maltophilia*, the most tested antibiotics for susceptibility were TMP-SMX (n = 356 [95.2%]), levofloxacin (n = 254 [67.9%]), and ceftazidime (n = 153 [40.9%]), and resistant isolates were reported for these antibiotics 4.5%, 4.3%, and 44.4% of the time, respectively (Figure 1). For the 282 unique patients with *S. maltophilia* BSI, the most frequently used antibiotics with possible activity against the pathogen were TMP-SMX (41.5%) and levofloxacin (39.4%), followed by ciprofloxacin (13.8%) and ceftazidime (12.4%). Combination therapy, with at least 2 active agents, was given in 32.6% of the cases (Table 2). A total of 62 patients (22%) did not receive any antibiotic with possible activity within the prespecified window of days –2 to +5 of index date. Among these patients, 15 (24%) did not receive treatment due to mortality prior to therapy or withdrawal of care. Other reported reasons for not initiating antibiotics active against *S. maltophilia* included administration of therapy outside of the prespecified time frame of study protocol, and no treatment per clinician judgment based on symptoms, source control, or other microbiologic data.

**Table 2.** Antimicrobial treatment for *S. maltophilia* BSI

Antibiotic(s)	N (%)
TMP-SMX	117 (41.5)
Levofloxacin	111 (39.4)
Ciprofloxacin	39 (13.8)
Ceftazidime	35 (12.4)
Tetracycline	18 (6.4)
Minocycline	15 (5.3)
Moxifloxacin	8 (2.8)
Polymyxins	4 (1.4)
Tigecycline	3 (1.1)
Combination (≥2 agents)	92 (32.6)
None	62 (22.0)

**Figure 2.**
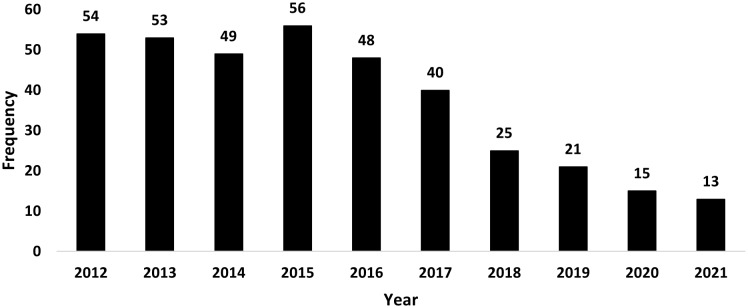
Frequencies of *S. maltophilia* positive blood cultures by year across 75 VA facilities.

**Figure 3.**
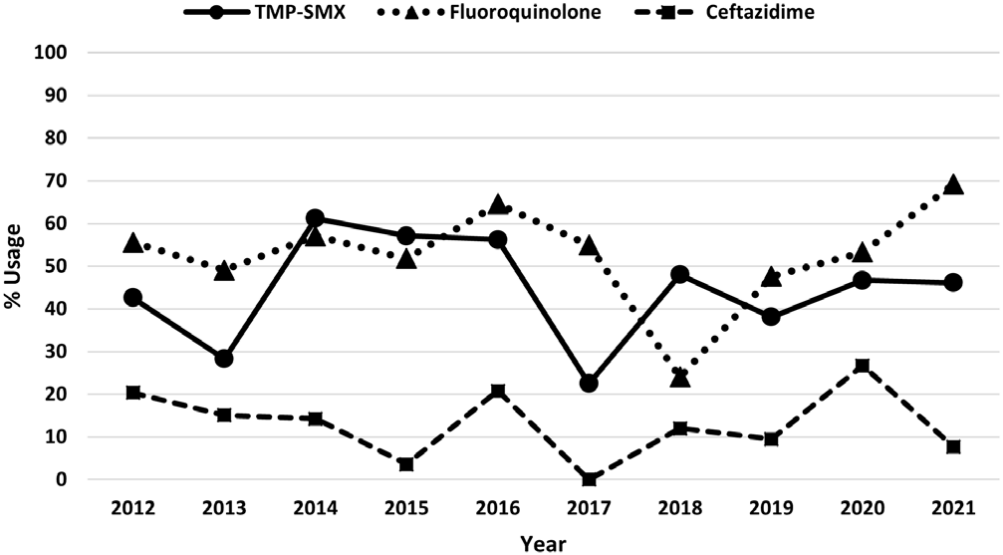
Trends in prescribing of the most commonly used agents for *S. maltophilia* BSI in Veterans from 2012 to 2021.

From 2012 to 2021, the frequency of *S. maltophilia* blood cultures were noted to decrease over time, with 14.4% in 2012 to 3.5% in 2021 (Figure 2). No significant trends were observed in TMP-SMX (IIR = 1.00, 95% CI 0.94–1.06, *p* = 0.99), fluoroquinolones (IRR = 0.99, 95% CI 0.94–1.05, *p* = 0.81), or ceftazidime (IIR = 0.94, 95% CI 0.83–1.05, *p* = 0.28) prescribing over this time frame (Figure 3).

## Discussion

TMP-SMX and levofloxacin were the most commonly prescribed antibiotics for *S. maltophilia* BSI, with no discernible trends in utilization over time. Despite TMP-SMX historically being considered the preferred therapy for *S. maltophilia*, the utilization of fluoroquinolones was similar to that of TMP-SMX. The demographics of the patients were predominantly male, white, and older. These characteristics were consistent with the national VA population statistics.^12^ Although 34% of patients had documentation of fluroquinolone use 90 days prior to the index date, resistance rates were similar in fluoroquinolones compared to TMP-SMX. The utilization of other potentially effective agents was low. The low usage of minocycline in this study (5.4% of patients) has been described elsewhere in a similar database study, possibly due to tetracyclines not being preferred in practice for the treatment of BSI.^11^ Although only a few isolates were tested for minocycline susceptibility, only 2.6% were reported to be resistant. No cases received cefiderocol, eravacycline, or the combination of ceftazidime-avibactam plus aztreonam, which is likely due to the relatively recent approval of these newer agents as well as the time frame of this study predating the IDSA Antimicrobial Resistance (AMR) guideline release. An overall downward trend was observed in the frequency of *S. maltophilia* BSI in the 10-year time frame studied with the majority of cases observed in southern geographical regions.

As specimens other than blood isolates of *S. maltophilia* may sometimes indicate colonization rather than true infection, this study exclusively focused on patients with *S. maltophilia* in the blood. Although clinical outcomes were not evaluated, the results were remarkable in that 22% of the patients studied did not receive antimicrobial treatment within the evaluated time frame active against the *S. maltophilia*. While about a quarter of these cases did not receive culture directed therapy due to mortality prior to therapy or withdrawal of care, there were also instances where patients, if asymptomatic, were monitored without receiving treatment specific for the *S. maltophila*. While the presence of gram-negative pathogens in the blood is not typically considered contamination, it is conceivable that the *S. maltophilia* was not treated in the setting of absence of symptoms.

Prior studies have also suggested that appropriate treatment is not consistently provided after *S. maltophilia* is detected in blood cultures. If treatment is provided, fluoroquinolones have been found to be prescribed more often than TMP-SMX. A retrospective observational study of Japanese medical facilities aimed to describe clinical characteristics of *S. maltophilia* BSI cases between 2007 and 2013.^13^ Out of a total of 44 cases, 36% were reported to have not received appropriate antimicrobial therapy, yet 69% of these patients survived. The most frequently administered antimicrobial class was fluoroquinolones, followed by minocycline and TMP-SMX (15, 10, and 3 cases, respectively). A more recent retrospective database study similarly evaluated patients with *S. maltophilia* BSI between 2010 and 2015 to describe patient, microbiologic, and treatment characteristics.^2^ Out of 486 unique cases, 95% and 84% of cultures were susceptible to TMP-SMX and fluoroquinolones, respectively, and utilization of levofloxacin was higher (48.9%) when compared to TMP-SMX (38.3%). In this study, 15% of patients did not receive appropriate definitive therapy.

Studies on *S. maltophilia* infections in Veterans are scarce overall, and even more limited specifically for BSI. National VA data from 2010 to 2018 demonstrated a significant decrease in *S. maltophilia* prevalence (by 5.4% per year) as well as a decrease in resistance to TMP-SMX over time.^13^ The authors theorized that the decreasing trend in cultures may be related to efforts in inpatient settings to improve infection control and antimicrobial stewardship across the health system. Although less than 10% of the hospital cultures (n = 10,513) were from the blood, the downtrend in prevalence of *S. maltophilia* was consistent with the results of our study, even when cultures from other body sites were included in the analysis (ie, urine, skin and soft tissue, or respiratory). The same authors evaluated treatment of *S. maltophilia* infections in the Veteran population and found that the most common antibiotic agents used over a 9-year period were piperacillin/tazobactam (39.7%), TMP-SMX (23.3%), and levofloxacin (23.2%), however these antimicrobials may have been directed toward other pathogens in the case of a polymicrobial culture.^11^ Interestingly, combination therapy was used in 16.6% of patients, 50% less than findings from the current study which evaluated more recent data.

Limitations to the current study include the relatively small sample size, despite the wide time frame reviewed and the inclusion of a large number of VA facilities, and the mostly male sample minimizing generalizability to females. Despite this, the current study adds to the limited data in the literature evaluating treatment trends specifically for *S. maltophilia* BSI. Our study did not consider variability in susceptibility testing across different VA facilities nor the potential advancements in laboratory methods that could have enhanced the detection and identification of organisms in blood cultures over the 10-year period, however, frequency of positive *S. maltophilia* blood cultures in the current study decreased over time. Considering the historical practice of evaluating blood culture results and antibiotic therapy for suspected bacteremia over a period of up to 5 days, along with the average time-to-positivity of *S. maltophilia* observed in previous studies, we established the inclusion criteria as antibiotic administration during −2 to +5 days from the index date.^14–20^ This time frame was chosen to encompass the majority of patients with positive cultures who received pathogen-directed therapy, however the incidence of those who had delays in culture positivity and treatment administration is unclear. Finally, due to the time frame evaluated, this study did not capture any patients who were treated with newly approved antimicrobials, as currently recommended in the IDSA guidelines, thus more recent data is needed to describe the uptake of these agents.

In conclusion, TMP-SMX and levofloxacin were the most commonly used antibiotics for the treatment of *S. maltophilia* BSI in Veterans during 2012–2021. Despite TMP-SMX being the preferred agent historically, its use was comparable to fluoroquinolones. The utilization of tetracyclines was low, and there was no utilization of newer antibiotic agents. Combination therapy was prescribed in only 33% patients contrary to the recent proposed approach of initiating combination therapy to increase the likelihood that at least one agent is effective against the organism.

As newer antimicrobial agents showing efficacy against *S. maltophilia* are introduced and guidance for managing *S. maltophilia* infections expands, we anticipate future treatment trends to be more diverse, incorporating a broader range of agents and favoring combination therapy over the traditional reliance on TMP-SMX and fluoroquinolones.

The selection of agents was appropriate in majority of cases as both TMP-SMX and levofloxacin can be effective for *S. maltophilia* infections. As the management of *S. maltophilia* evolves, future trends are expected to incorporate newer antimicrobials, such as cefiderocol, or higher usage of combination therapy. TMP-SMX and levofloxacin are anticipated to remain as viable options for *S. maltophilia* infections given limited effective therapies.

## Research transparency and reproducibility

We are committed to collaborating and sharing these data to maximize their value to improve Veterans and others’ health and health care, to the greatest degree consistent with current Veterans Administration regulations and policy. We can provide access to the programming code used to identify the sample and conduct analyses. We cannot provide a link to the database directly as it would compromise patients’ anonymity, and permissions from VA are needed to obtain the data.

## References

[1] Kullar R , Wenzler E , Alexander J , Goldstein EJC. Overcoming *Stenotrophomonas maltophilia* resistance for a more rational therapeutic approach. Open Forum Infect Dis 2022;9:ofac095. doi: 10.1093/ofid/ofac095. 35415194 PMC8992361

[2] Cai B , Tillotson G , Benjumea D , Callahan P , Echols R. The burden of bloodstream infections due to *Stenotrophomonas maltophilia* in the United States: a large, retrospective database study. Open Forum Infect Dis 2020;7:ofaa141. doi: 10.1093/ofid/ofaa141. 32462047 PMC7240339

[3] Brooke JS. Advances in the microbiology of *Stenotrophomonas maltophilia* . Clin Microbiol Rev 2021;34:e0003019. doi: 10.1128/CMR.00030-19. PMC8262804 34043457

[4] Safdar A , Rolston K V. *Stenotrophomonas maltophilia*: changing spectrum of a serious bacterial pathogen in patients with cancer. Clin Infect Dis 2007;45:1602–1609. 18190323 10.1086/522998

[5] Anđelković M V. , Janković SM , Kostić MJ , et al. Antimicrobial treatment of *Stenotrophomonas maltophilia* invasive infections: systematic review. J Chemother 2019;31:297–306. 31130079 10.1080/1120009X.2019.1620405

[6] Nicodemo AC , Paez JIG. Antimicrobial therapy for *Stenotrophomonas maltophilia* infections. Eur J Clin Microbiol Infect Dis 2007;26:229–237. 17334747 10.1007/s10096-007-0279-3

[7] JMI Laboratories. Activity of Antimicrobial Agents Tested against 4,187 *Stenotrophomonas maltophilia* Isolates in the SENTRY Program.; 2022. sentry-mvp.jmilabs.com. Accessed October 30, 2022.

[8] Gibb J , Wong DW. Antimicrobial treatment strategies for *Stenotrophomonas maltophilia*: a focus on novel therapies. Antibiotics 2021;10:1226. doi: 10.3390/antibiotics10101226. 34680807 PMC8532924

[9] Sader HS , Jones RN. Antimicrobial susceptibility of uncommonly isolated non-enteric gram-negative bacilli. Int J Antimicrob Agents 2005;25:95–109. 15664479 10.1016/j.ijantimicag.2004.10.002

[10] Tamma PD , Aitken SL , Bonomo RA , Mathers AJ , van Duin D , Clancy CJ. Infectious diseases society of America 2023 guidance on the treatment of antimicrobial resistant gram-negative infections. Clin Infect Dis 2023;74:2089–2114. 10.1093/cid/ciad428 37463564

[11] Charlson ME , Pompei P , Ales KL , MacKenzie CR. A new method of classifying prognostic comorbidity in longitudinal studies: development and validation. J Chronic Dis 1987;40:373–383. 3558716 10.1016/0021-9681(87)90171-8

[12] Reports - National Center for Veterans Analysis and Statistics. Published September 30, 2021. https://www.va.gov/vetdata/report.asp. Accessed April 30, 2024.

[13] Ebara H , Hagiya H , Haruki Y , Kondo E , Otsuka F. Clinical characteristics of *Stenotrophomonas maltophilia* bacteremia: a regional report and a review of a Japanese case series. Intern Med 2017;56:137–142. 28090041 10.2169/internalmedicine.56.6141 PMC5337456

[14] Pollack LA , Srinivasan A. Core elements of hospital antibiotic stewardship programs from the centers for disease control and prevention. Clin Infect Dis 2014;59:S97–100. 25261548 10.1093/cid/ciu542 PMC6521960

[15] Lambregts MMC , Bernards AT , van der Beek MT , Visser LG , de Boer MG. Time to positivity of blood cultures supports early re-evaluation of empiric broad-spectrum antimicrobial therapy. PLoS One 2019;14:e0208819. 30601829 10.1371/journal.pone.0208819 PMC6314566

[16] Reimer LG , Wilson ML , Weinstein MP. Update on detection of bacteremia and fungemia. Clin Microbiol Rev 1997;10:444–465. 9227861 10.1128/cmr.10.3.444 PMC172929

[17] Gajdács M , Urbán E. Epidemiological trends and resistance associated with *Stenotrophomonas maltophilia* bacteremia: A 10-year retrospective cohort study in a tertiary-care hospital in hungary. Diseases 2019;7:41. doi: 10.3390/diseases7020041. 31159258 PMC6631814

[18] Lakatos B , Jakopp B , Widmer A , et al. Evaluation of treatment outcomes for *Stenotrophomonas maltophilia* bacteraemia. Infection 2014;42:553–558. 24627266 10.1007/s15010-014-0607-3

[19] Ransom EM , Alipour Z , Wallace MA , Burnham CAD. Evaluation of optimal blood culture incubation time to maximize clinically relevant results from a contemporary blood culture instrument and media system. Simner PJ, ed. J Clin Microbiol 2021;59:e02459-20. doi: 10.1128/JCM.02459-20. 33239377 PMC8106720

[20] Reimer LG , Wilson ML , Weinstein MP. Update on detection of bacteremia and fungemia. Clin Microbiol Rev 1997;10:444–465. 9227861 10.1128/cmr.10.3.444 PMC172929

